# Fully Automated Delineation of Gross Tumor Volume for Head and Neck Cancer on PET-CT Using Deep Learning: A Dual-Center Study

**DOI:** 10.1155/2018/8923028

**Published:** 2018-10-24

**Authors:** Bin Huang, Zhewei Chen, Po-Man Wu, Yufeng Ye, Shi-Ting Feng, Ching-Yee Oliver Wong, Liyun Zheng, Yong Liu, Tianfu Wang, Qiaoliang Li, Bingsheng Huang

**Affiliations:** ^1^School of Biomedical Engineering, Health Science Center, Shenzhen University, Shenzhen, China; ^2^Medical Physics and Research Department, Hong Kong Sanatorium & Hospital, Happy Valley, Hong Kong; ^3^Department of Radiology, Guangzhou Panyu Central Hospital, Guangzhou, China; ^4^Department of Radiology, First Affiliated Hospital, Sun Yat-sen University, Guangzhou, China; ^5^University of Southern California, Los Angeles, USA; ^6^Intensive Care Unit, Southern Medical University Shenzhen Hospital, Shenzhen, China

## Abstract

**Purpose:**

In this study, we proposed an automated deep learning (DL) method for head and neck cancer (HNC) gross tumor volume (GTV) contouring on positron emission tomography-computed tomography (PET-CT) images.

**Materials and Methods:**

PET-CT images were collected from 22 newly diagnosed HNC patients, of whom 17 (Database 1) and 5 (Database 2) were from two centers, respectively. An oncologist and a radiologist decided the gold standard of GTV manually by consensus. We developed a deep convolutional neural network (DCNN) and trained the network based on the two-dimensional PET-CT images and the gold standard of GTV in the training dataset. We did two experiments: Experiment 1, with Database 1 only, and Experiment 2, with both Databases 1 and 2. In both Experiment 1 and Experiment 2, we evaluated the proposed method using a leave-one-out cross-validation strategy. We compared the median results in Experiment 2 (GTVa) with the performance of other methods in the literature and with the gold standard (GTVm).

**Results:**

A tumor segmentation task for a patient on coregistered PET-CT images took less than one minute. The dice similarity coefficient (DSC) of the proposed method in Experiment 1 and Experiment 2 was 0.481∼0.872 and 0.482∼0.868, respectively. The DSC of GTVa was better than that in previous studies. A high correlation was found between GTVa and GTVm (*R* = 0.99, *P* < 0.001). The median volume difference (%) between GTVm and GTVa was 10.9%. The median values of DSC, sensitivity, and precision of GTVa were 0.785, 0.764, and 0.789, respectively.

**Conclusion:**

A fully automatic GTV contouring method for HNC based on DCNN and PET-CT from dual centers has been successfully proposed with high accuracy and efficiency. Our proposed method is of help to the clinicians in HNC management.

## 1. Introduction

Head and neck cancer (HNC) is a type of cancer originating from the tissues and organs of the head and neck with high incidence in Southern China [1]. Radiation therapy (RT) is one of the most effective therapies, which relies heavily on the contouring of tumor volumes on medical images. However, it is time-consuming to delineate the tumor volumes manually. Besides, the manual delineation is subjective, and the accuracy depends on the experience of the treatment planner. Compared to manual delineation, automatic segmentation can be relatively objective. Nowadays, there have been studies reporting the automatic segmentation of tumor lesions on magnetic resonance images of HNC using different methods [2–10].

Positron emission tomography-computed tomography (PET-CT) has played an important role in the diagnosis and treatment of HNC, providing both anatomical and metabolic information about the tumor. The automatic or semiautomatic segmentation of tumor lesions on PET-CT or PET images of HNC has been reported, using machine-learning (ML) methods such as k-nearest neighbor (KNN) [11, 12], Markov random fields (EM-MRFs) [13], adaptive random walker with k-means (AK-RW) [14], decision tree algorithm [15], and active surface modeling [16]. The segmentation of tumor lesions on the coregistered PET and CT images has shown better results than those on solely PET or CT images [17, 18]. However, the application of PET-CT has increased the amount and the complexity (multimodality) of the image data. Also, to propose a robust and practical ML-based automatic segmentation method, it is often necessary to train and test the method with heterogeneous image data from multicenter [19], which makes the training and testing of ML systems more challenging.

Compared to the traditional ML methods, deep learning (DL) allows extracting the features automatically instead of subjective feature extraction and selection in conventional ML techniques, which may be more appropriate for automatic segmentation in multimodality data and multicenter data. DL can easily recognize the intrinsic features of the data [20]. DL techniques, such as stacked denoising autoencoder (SDAE) [21] and convolutional neural network (CNN) [22–24], have been used in tumor segmentation successfully with improved accuracy.

No studies have been reported to apply the deep convolutional neural network (DCNN) in the automatic GTV delineation for HNC patients on PET-CT images. In our study, we proposed an automatic method of GTV delineation for RT planning of HNC based on DL and dual-center PET-CT images, aiming to improve the efficiency and accuracy.

## 2. Materials and Methods

In brief, our methodology included the contouring of the gold standard, training and testing of the DL model, and evaluating the performance of our trained model. After reviewing the MRI, CT, and PET images, an oncologist and a radiologist decided the contouring of GTV by consensus which was treated as the gold standard in the following training and testing of our method. We developed a deep convolutional neural network (DCNN) for HNC tumor lesion segmentation, and then we trained the network based on the PET-CT images and the gold standard of GTV in the training dataset. In the testing step, we input the testing dataset to the network, and it automatically contoured the GTV. To test the accuracy of this automated method, we compared the results of our method with those of other methods in the literature and with the gold standard.

### 2.1. Structure of Our DCNN Model

**Figure 1:**
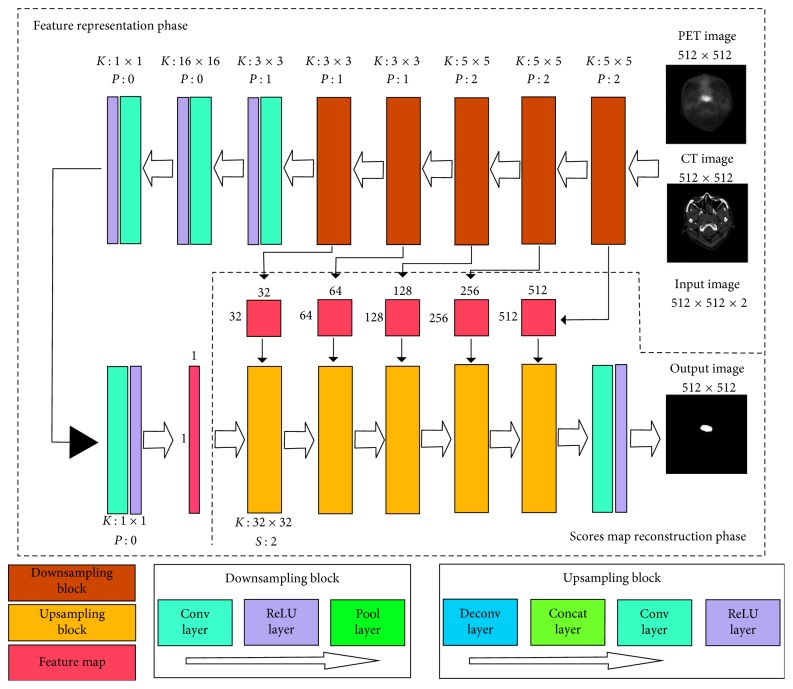
Architecture of the proposed CNN model. The proposed network includes two phases: feature representation phase and scores map reconstruction phase. The feature representation phase is composed of 5 downsampling blocks (conv-ReLU-pool layer), 4 convolution layers, and 4 ReLU layers. The scores map reconstruction phase consists of 5 upsampling blocks (deconv-concat-conv-ReLU layer), a convolution layer, and an ReLU layer. K, filter size; P, zero padding; S, stride.

Inspired by the fully convolutional network [25] and U-net [26], we designed a DCNN model for GTV delineation. The structure of our proposed DCNN model is shown in Figure 1. This network consisted of two stages: feature representation phase and scores map reconstruction phase.

#### 2.1.1. Feature Representation Phase

The main purpose of the feature representation phase was to extract the feature information of PET images and CT images, by combining the low-level features to represent the high-level features with semantic information. The feature representation phase contained 5 downsampling blocks, 4 convolution (conv) layers, and 4 rectifier linear unit (ReLU) layers (Figure 1). A downsampling block included a convolution layer, an ReLU layer, and a pooling (pool) layer. The first convolution layer was to extract the low-level features of PET images and CT images, respectively, by filters of 5 × 5 voxels and to fuse them together. We were able to fuse the features because the PET and CT images were input simultaneously with the same gold standard. We in the next 4 convolution layers applied the convolutions for the permutation and combination of the low-level features to obtain more high-level features with semantic information. In all the 5 downsampling blocks, the convolution layers were followed by a pooling layer. We applied pooling with 2 × 2 filters and 2 strides which decreased the length and width of the feature map by 50%. Thus, it could reduce the number of connection parameters and the computational time and provided the position invariance and more global information. The use of unaltered filters on a smaller image may contribute to the larger local receptive fields, and these enlarged local receptive fields could extract more global features. After each convolution layer, we used an ReLU layer as an activation layer to increase the nonlinearity of our network and to accelerate the convergence.

The length and width of the feature maps were reduced by 50% after a downsampling block. After the feature map size was reduced to 16 × 16, it was then connected with a convolution layer with 16 × 16 filters. It means that every neuron in the following layer was connected with all the neurons in the previous layer to imitate the fully connected layer in the traditional classification network. The size of the feature maps was 1 × 1 pixel after this convolution layer. Then, we used 2 convolution layers with 1 × 1 filters for the permutation and combination of these features to obtain more abstract information. The finally acquired 1 × 1 scores maps were used as the input in the scores map reconstruction phase.

#### 2.1.2. Scores Map Reconstruction Phase

The main purpose of the scores map reconstruction phase was to reconstruct the scores map into the same size of input images by upsampling. This reconstruction phase consisted of 5 upsampling blocks, a convolution layer, and an ReLU layer. An upsampling block was composed of a deconvolution (deconv) layer, a concatenation (concat) layer, a convolution layer, and an ReLU layer. The deconvolution layer was designed for upsampling. The first deconvolution layer reconstructed the 1 × 1 scores map to 32 × 32 by 32 × 32 filters. However, we found that deconvolution would cause the loss of the high-resolution information in images. To overcome this problem, we utilized the concatenation layer to fuse the feature maps in the previous pooling layers or convolution layers with the current feature maps in the deconvolution layer. We believed that these skip-layer designs could capture more multiscale contextual information and improve the accuracy of segmentation. To fuse the low- and high-resolution information pixel by pixel, we set the filters of all the following convolution layers at 1 × 1.

With all the upsampling blocks, we finally reconstructed the scores maps to an output image with a size of 512 × 512, the same as in the input PET or CT images. In order to optimize the network, we estimated the loss by calculating the Euclidean distance between the gold standard and the reconstructed tumor lesions [27, 28]. Then, the parameters of the network were iterated and renewed by backpropagation from the loss. In our experiment, we decided to use the Euclidean distance to estimate the loss because it had shown better performance than the cross entropy loss that was used in some other studies of Ronneberger et al. [26].

### 2.2. Training of Our DCNN Model

#### 2.2.1. Data Preprocessing

Newly diagnosed HNC patients were retrospectively recruited from two centers: 17 (13 males, 4 females; 31∼68 years old) from the First Affiliated Hospital, Sun Yat-sen University (center 1); and 5 (all males; 44∼63 years old) from Sun Yat-sen University Cancer Center (center 2). The ethics committee waived the necessity to obtain informed written consent from the patients. The PET-CT scans in both centers were from the top of the skull to the shoulder. Acquisition time of PET for each bed position was 2.5 minutes. The patients from center 1 were scanned with Discovery STE (GE Healthcare, Milwaukee, USA); the spatial resolution and image matrix of most CT images were 0.49 × 0.49 × 2.5 mm^3^ and 512 × 512 × 63, respectively, while the spatial resolution and image matrix of the PET images were 1.56 × 1.56 × 3.27 mm^3^ and 256 × 256 × 47, respectively. The PET scan in center 1 was acquired in 3-dimensional mode and reconstructed using the ordered-subset expectation maximization iterative algorithm. The patients from center 2 were scanned with Discovery 690 PET-CT scanners (GE Healthcare, Milwaukee, USA); the spatial resolution and image matrix of the CT images were 0.59 × 0.59 × 3.27 mm^3^ and 512 × 512 × 47, respectively, while the spatial resolution and image matrix of PET images were 1.17 × 1.17 × 3.27 mm^3^ and 256 × 256 × 47, respectively. In center 2, the PET scanning was acquired in 3-dimensional mode and reconstructed using the VPFXS reconstruction method.

To make use of the information of both the PET image and the CT image, we performed coregistration of PET to CT images by sampling the PET images using linear interpolation in SPM8 (Wellcome Department of Imaging Neuroscience, London, United Kingdom). Finally, we had 934 samples (one sample includes one slice of the CT image and one coregistered slice of the PET image, both with a matrix size of 512 × 512) for the 17 patients from center 1 as Database 1 and 200 samples for the 5 patients from center 2 as Database 2.

The primary GTVs were manually outlined by an experienced radiologist and double-checked by an experienced oncologist on the registered PET/CT with reference to MRI, PET, and CT images using the ITK-SNAP software (http://www.itksnap.org) [29]. The resultant GTV contouring was used as the gold standard in training and testing of our proposed model and for the comparisons with automatic segmentation in terms of their volume and geometrical overlap. Specifically, we discarded the images in which the tumor size was smaller than 0.5 cm^2^ (in the 2-dimensional images) by considering the partial-volume effect (PVE) in the PET image, suggested by the radiologist. PVE could affect the imaging accuracy of small tumor lesions whenever the tumor size is less than 3 times the full width at half maximum (FWHM) of the reconstructed image resolution [30].

We performed two experiments with our data. In Experiment 1, we evaluated the proposed method using only the data in Database 1. We evaluated the proposed method using a leave-one-out cross-validation (LOOCV) strategy, leaving the images of one patient for testing and the images of all other patients for training. To balance the positive and negative samples in the training dataset, we selected all the slices with tumor lesions as positive samples and randomly selected the same number of slices without tumor lesions as positive samples. To satisfy the need of huge training data in DL, we augmented the training dataset to nearly 15,000 samples by rotating the images, horizontal mirroring, changing the contrast, and image scaling. In Experiment 2, we used the two databases (1134 samples) and augmented the training dataset to nearly 18,000 samples and also evaluated the method by using the LOOCV strategy similarly. Before training and testing in both Experiments 1 and 2, all data were normalized by performing min-max normalization.

#### 2.2.2. Network Training

The training of the whole network was composed of three stages. At the first stage, we obtained an output image after the third upsampling block (Figure 1), and the size of the output image was 128 × 128. In the second stage, which was initialized by the network parameters in the first stage, a 256 × 256 scores map was obtained. Finally, we based on the network parameters in the second stage trained the whole network, and the scores maps were used to reconstruct an output image with a size of 512 × 512 (the same as the size of the input PET or CT images).

The model was trained by using an Adam optimizer for 200,000 iterations with a fixed learning rate of 0.00001. We used a GPU NVIDIA GeForce GTX 1080 Ti equipped on an Intel Xeon E5-2650 2.30 GHz × 16 machine and the DL framework Keras for training [31]. The whole training procedure took about 24 hours.

### 2.3. Performance Evaluation of Our DCNN Method

#### 2.3.1. Evaluation of Automatic GTV Delineation Performance

After the successful training of the DCNN model, we used the testing dataset to evaluate the segmentation performance of our method by calculating the dice similarity coefficient (DSC) as follows:

(1) $$\begin{array}{c} \text{DSC}=\frac{2\text{TP}}{\text{FP}+2\text{TP}+\text{FN}}, \end{array}$$

where true positive (TP) denotes the correctly identified tumor area, false positive (FP) denotes the normal tissue that is incorrectly identified as tumor, and false negative (FN) denotes the tumor area that is incorrectly predicted as normal tissue. DSC describes the overlap between the gold standard and the automatic segmentation result.

#### 2.3.2. Comparison with Other Methods in the Literature

To instigate the improvement of our method, we also compared our results with the previous studies. We tried to apply these previous methods on our database; however, the performance was all lower than the published results. Hence, we directly compared our results with those in these publications, in terms of DSC. Although they may not be reasonably comparable, these comparisons to some extent provide insights about how our method outperformed the similar studies. Note that for a fair comparison, we used the results of median performance in Experiment 2 for the comparison.

#### 2.3.3. Comparison with the Gold Standard of GTV

Although we repeated our experiments for several times, for a fair comparison, we used the results of median performance in Experiment 2 with dual-center data for the comparison, and the results were recorded as GTVa. The gold standard by manual contouring was recorded as GTVm. Pearson's correlation was performed between GTVa and GTVm. To further evaluate the accuracy of GTVa against GTVm, we calculated mean surface distance (MSD), sensitivity, and precision as follows:

(2) $$\begin{array}{c} \text{MSD}\left(X,Y\right)=\frac{1}{2}\cdot \left[\frac{1}{N}\overset{N}{\underset{i=1}{\sum }}\underset{j=1,\ldots ,M}{\min}\left\|y_{i}^{1}-y_{j}^{2}\right\|+\frac{1}{M}\overset{M}{\underset{j=1}{\sum }}\underset{i=1,\ldots ,N}{\min}\left\|y_{i}^{1}-y_{j}^{2}\right\|\right],\\ \text{sensitivity}=\frac{\text{TP}}{\text{TP}+\text{FN}},\\ \text{precision}=\frac{\text{TP}}{\text{TP}+\text{FP}}, \end{array}$$

where *X* and *Y* denote the boundary of autosegmentation and the gold standard, respectively (*y*_*i*_^1^, *i*=1,…, *N* ∈ *X* is the boundary points of *X*; *y*_*j*_^2^, *j*=1,…, *M* ∈ *Y* is the boundary points of *Y*, respectively). MSD describes the mean Euclidean distance between GTVa and GTVm along their boundaries. Sensitivity describes how much the overlap of GTVa and GTVm was included in GTVm. Precision describes how much the overlap of GTVa and GTVm was included in GTVa. The absolute difference between GTVa and GTVm was also estimated by calculating GTVa − GTVm [32].

## 3. Results

### 3.1. Automatic GTV Delineation Performance

**Figure 2:**
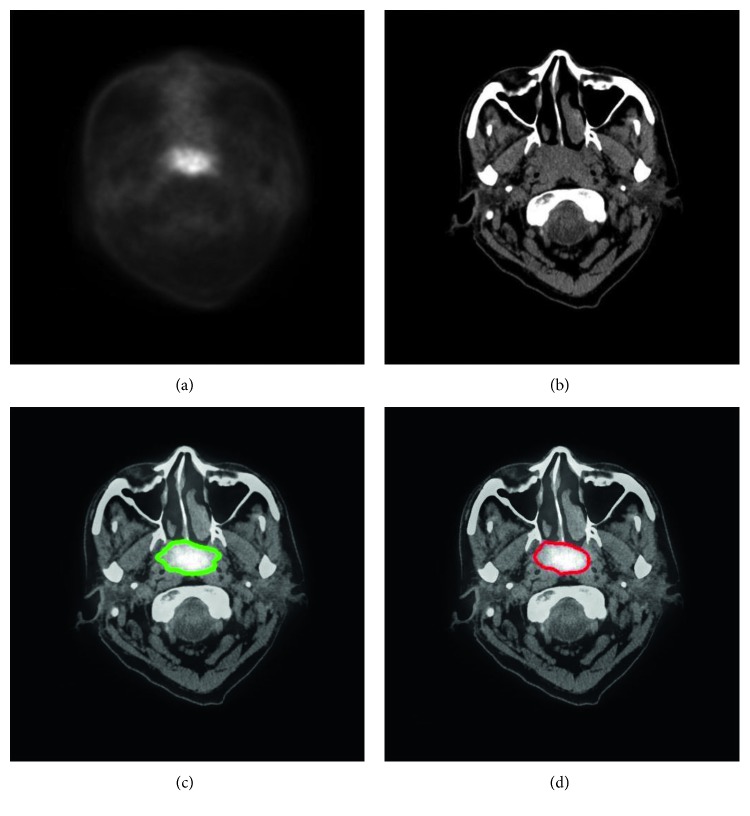
An example of HNC tumor segmentation with high accuracy. The dice similarity coefficient (DSC) was 0.943. (a) PET image coregistered with CT. (b) CT image. (c) Automatic segmentation result presented on the fused PET-CT image (green line). (d) Gold standard of gross tumor volume drawn on the fused PET-CT image (red line).

**Figure 3:**
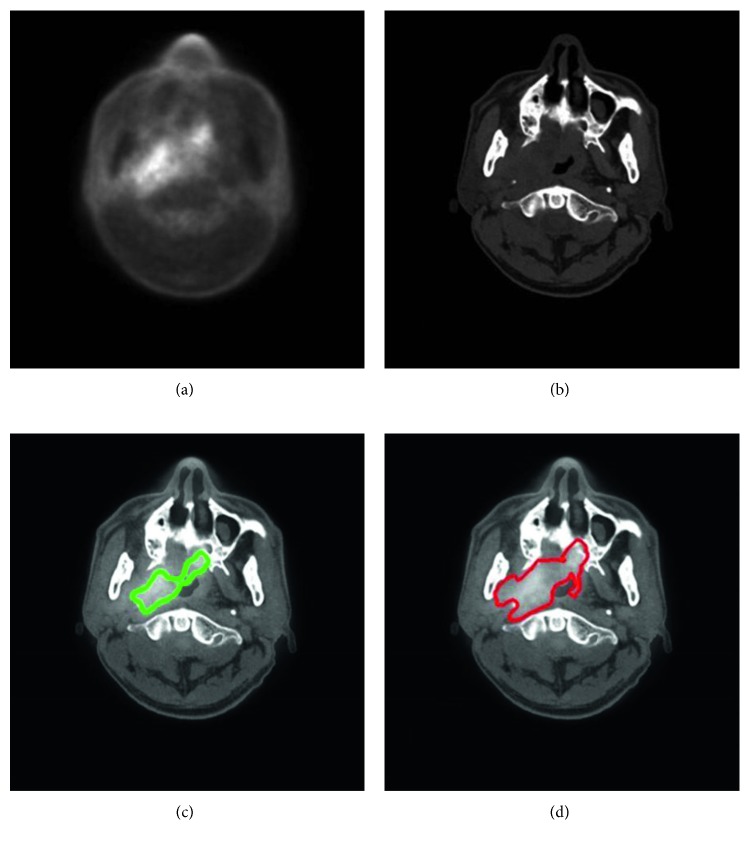
A typical example of HNC tumor segmentation with low accuracy. The dice similarity coefficient (DSC) was 0.610. (a) PET image coregistered to CT. (b) CT image. (c) Automatic segmentation result presented on the fused PET-CT image (green line). (d) Gold standard of gross tumor volume, drawn on the fused PET-CT image (red line).

**Figure 4:**
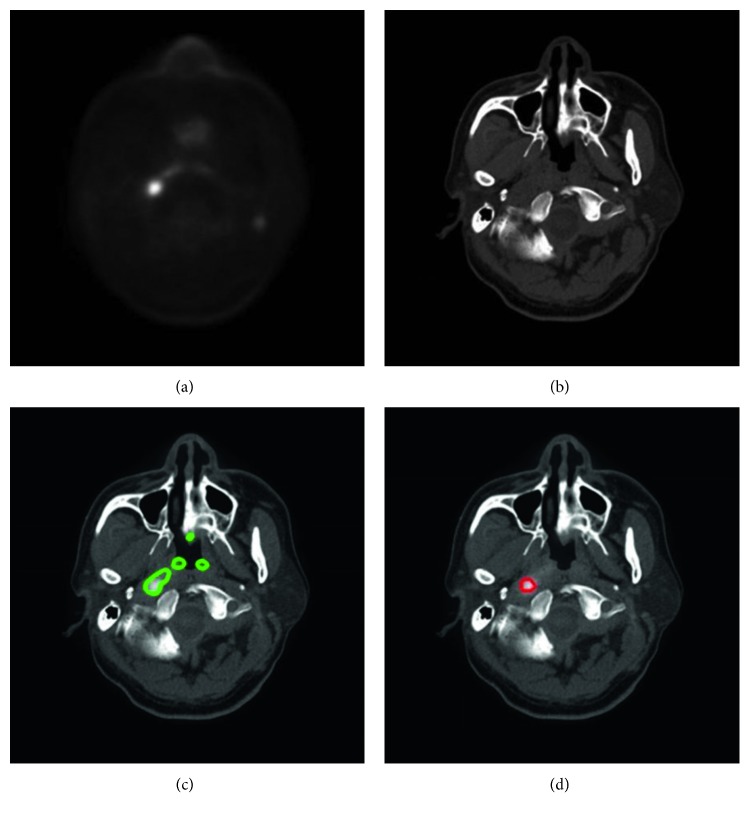
Another typical example of HNC tumor segmentation with low accuracy. The dice similarity coefficient (DSC) was 0.408. (a) PET image coregistered to CT. (b) CT image. (c) Automatic segmentation result presented on the fused PET-CT image (green line). (d) Gold standard of gross tumor volume, drawn on the fused PET-CT image (red line).

**Figure 5:**
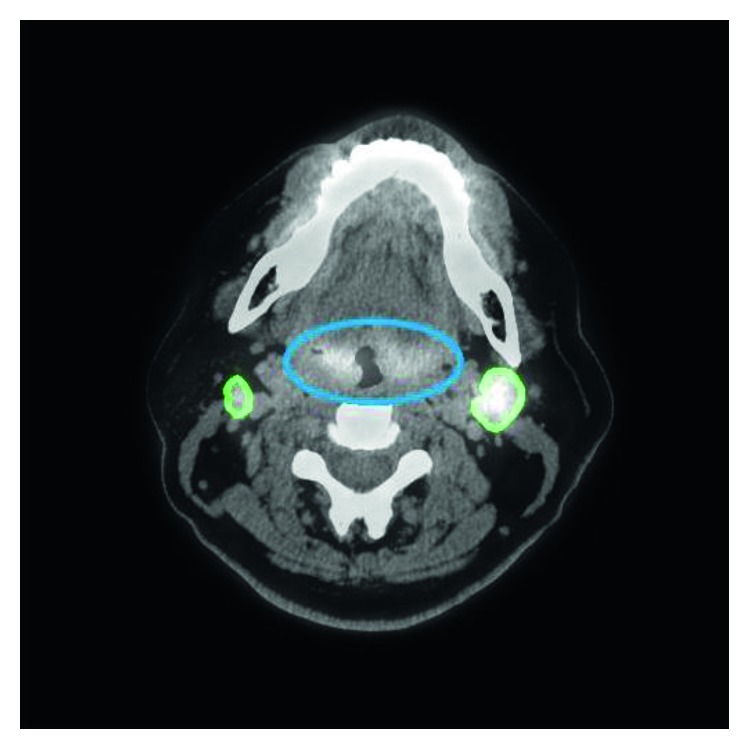
An example of inflammation. The inflammation region, which is marked by a blue line, looks similar to the tumor and so may be difficult to distinguish by visual assessment. However, it was recognized correctly by our proposed method (green line). The dice similarity coefficient (DSC) of this tumor was 0.848.

With our trained model, a tumor segmentation task for a sample (a coregistered PET image and a CT image, two-dimensional) took about 0.28 seconds; thus, for an HNC patient with around 50 slices of coregistered PET/CT images, our method took about 14 seconds for GTV segmentation. An example of segmentation with high accuracy is shown in Figure 2, in which the DSC was 0.943. Two typical examples of the poor results and their corresponding PET images are shown in Figures 3 and 4, in which the DSC was 0.610 and 0.408, respectively. As shown in Figure 5, the region marked by the blue circle with high metabolism was actually an inflammation region, which looks very similar to the tumor lesions. Our trained model was able to learn the difference between the inflammation regions and the tumor lesions and correctly recognized this as a nontumor region.

The median DSC in Experiment 1 of 17 patients (Database 1) was 0.762 (range, 0.481∼0.872). The median DSC in Experiment 2 of 22 patients (Database 1 + Database 2) was 0.785 (range, 0.482∼0.868), 0.783 (range, 0.482∼0.868) for Database 1 alone, and 0.793 (range, 0.528∼0.863) for Database 2 alone. The segmentation results in Experiment 2 were recorded as GTVa and used for the following comparisons.

### 3.2. Comparison with Other Methods in the Literature

The results of previous studies about HNC segmentation based on PET-CT are shown in Table 1. The mean DSC of our method in Experiment 2 for 22 patients was 0.736. Stefano et al. [14] achieved a high DSC of 0.848; however, their method was on PET images only and was semiautomatic.

### 3.3. Comparison with the Gold Standard of GTV

**Figure 6:**
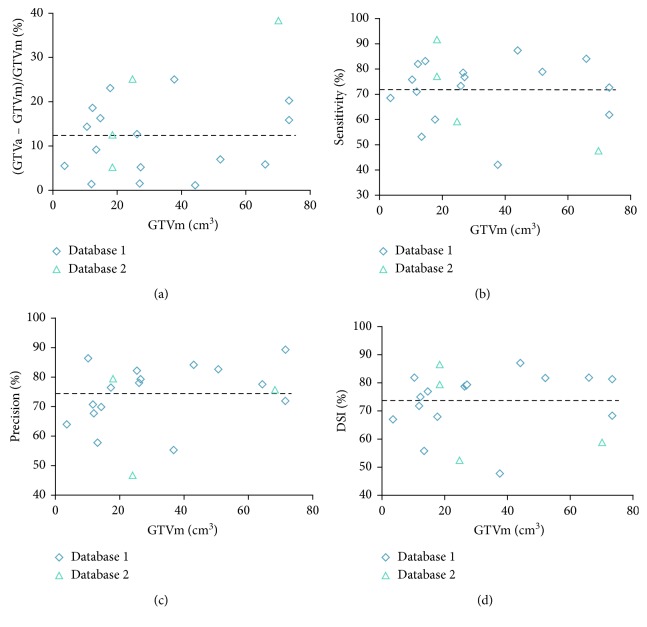
Comparisons between GTVm and GTVa. GTVm, the gross tumor volume by manual delineation (gold standard). GTVa, the gross tumor volume of automatic segmentation by the proposed method. Each point represents a patient. DSC, dice similarity coefficient. (a) Difference between GTVm and GTVa. Sensitivity (b), precision (c), and DSC (d) of GTVa (the average values are depicted as a thick dashed line).

Pearson's correlation showed a high correlation between GTVa and GTVm (*R* = 0.99, *P* < 0.001) for these 22 patients. The detailed comparison between GTVa and GTVm is shown in Table 2 and Figure 6. The mean volume difference (%) between GTVa and GTVm was 12.4% ± 9.8% with a 95% confidence interval (CI) of −6.7%∼31.6%. The average DSC, sensitivity, precision, and MSD of all patients were 0.736 ± 0.110 (95% CI, 0.521∼0.951), 0.720 ± 0.128 (95% CI, 0.468∼0.973), 0.761 ± 0.111 (95% CI, 0.543∼0.978), and 4.7 ± 3.4 mm (95% CI, −1.8∼11.2 mm), respectively.

## 4. Discussion

We proposed an HNC automated GTV contouring method based on DL and PET-CT images, with encouraging segmentation results. Most of the studies on HNC delineation were based on PET images only [14–17], in which the anatomical information was insufficient due to the low spatial resolution compared to CT or MRI [13]. Yang et al. [13] achieved similar segmentation accuracy (DSC = 0.740); however, their method was based on three modalities (PET, CT, and MRI). The methods of Stefano et al. [14] and Song et al. [17] were all semiautomatic. Berthon et al. [15] reported a higher accuracy of 0.77; however, their gold standard for performance evaluation incorporated the information of automatic segmentation results. Compared to these studies [13–17] with the data from one center only, our proposed method shows stable performance on dual-center data. To summarize, our proposed method has shown relatively high accuracy and is fully automatic, making use of both the metabolic and anatomic information.

Either in Experiment 1 or in Experiment 2, the performance was high and stable for Database 1. This may suggest that the proposed DCNN model was effective and robust. Note that in Experiment 2, the DSC was higher than that in Experiment 1. This may be because with more samples, more features can be learned by our DCNN model, and thus, the segmentation accuracy could be improved. However, we also in Experiment 2 observed that the accuracy for Database 2 was lower than that for Database 1. The reason may be that the features were somehow different between these two databases. The features learned from Databases 1 and 2, mainly from Database 1, were probably not suitable enough to be applied to Database 2. Note that with only 22 patients, we already achieved such good performance of automatic contouring. However, we may recruit more data to further verify the robustness of our model.

The image features are critical in machine-learning-based segmentation tasks. We used the multimodality images, namely, PET and CT images, as the input of our DCNN model, and this may improve the segmentation than with PET or CT images alone. This finding echoed the results reported in the study of Song et al. [17] or Bagci et al. [18]. As shown in Figure 2, since the metabolism is significantly different between tumor regions and normal tissues, the contrast of the tumor region to the adjacent tissues is high; thus, the location of tumor is easily detected in PET images. However, the spatial resolution of PET images is low; thus, the tumor boundary is unclear in PET images. In CT images with higher spatial resolution, the anatomical information is more sufficient for detecting the boundary of tumors. By using both PET and CT images, our method extracted and combined both metabolic and anatomical information as the efficient features for more accurate segmentation.

**Figure 7:**
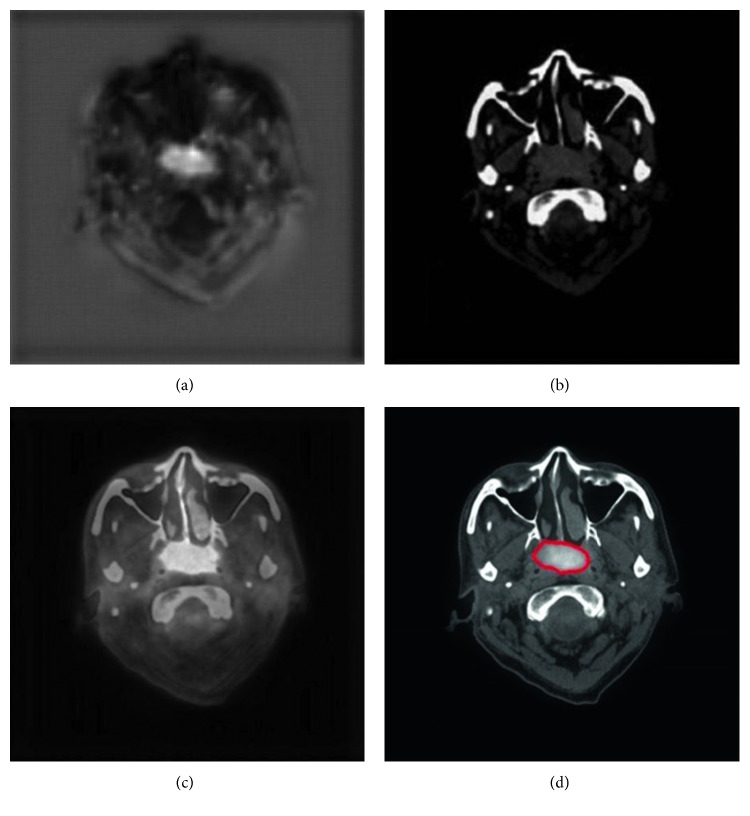
An example of the feature maps in the fourth upsampling block. (a) A low-resolution scores map after the fourth deconvolution layer in the reconstruction phase. (b) A high-resolution feature map after the fourth pooling layer in the feature representation phase. (c) The scores map after the fusion of low- and high-resolution maps. (d) The gold standard of gross tumor volume of the example (red line).

The DL technique we used to extract the features has shown more advantages than traditional machine-learning methods (Table 1). As shown in Figure 5, the region marked by the blue circle with high metabolism was actually an inflammation region, which looks very similar to the tumor lesions. The inexperienced clinicians may incorrectly consider this region as a tumor lesion, while our trained model was able to learn the difference between these inflammation regions and the tumor lesions and correctly recognized this as a nontumor region. Such an example showed that our DCNN method can extract the intrinsic features of tumor lesions and finally achieve better GTV contouring results. Besides, we used a skip-layer architecture for the fusion of the feature maps at the feature representation phase and scores map reconstruction phase, which can be another technical improvement in our method. As shown in Figure 7, although the semantic information of the features in the feature representation phase was worse than that of the features in the scores map reconstruction phase, it could help fix the problem of information loss in the reconstruction procedure. Compared to the feature map fusion method used by Long et al. [25], our method successfully incorporated the more useful features during the process of feature fusion. We believed that this fusion improves the accuracy of segmentation by using the skip-layer architecture.

The comparisons between GTVa and GTVm (Figure 6 and Table 2) indicated that GTVa was similar and close to GTVm. However, there were still some shortcomings in our automatic method. Firstly, the GTVa was unsatisfactory in some tumors. As shown in Figure 3, the tumor in the PET image was large, but the boundary was unclear; thus, part of the tumor was incorrectly identified as normal tissue. As shown in Figure 4, the low metabolism region, which was within the region where tumor lesions were often seen in some other patients, was incorrectly detected as tumor lesions. As shown in Figure 6(a), two patients showed a large difference between GTVm and GTVa. The tumors of these two patients were large with lots of lymphatic metastasis. This kind of tumor was few in our database; thus, our method failed to learn the features of these kinds of tumors. Secondly, we discarded the images in which the tumor size was smaller than 0.5 cm^2^ (in the 2-dimensional images) because such tumor lesions were difficult to detect in PET images by visual assessment. In addition, the imaging accuracy of small tumor lesions could be affected due to PVE. Thus, the performance of our method for such small tumors remains unclear.

Our results may be improved in future studies in the following aspects. Firstly, more data should be recruited for training a better model and to test-retest the performance. Especially, the data from different centers should be better balanced. Also, the MRI images may be employed as they provide better soft tissue contrast and may improve the performance. Secondly, in the training and testing, only the 2-dimensional images were used and the volumetric information was abandoned. We would carefully improve the network architecture and also adjust the training parameters for better segmentation results. Finally, for successful application of our method in the radiotherapy of HNC, the automatic contouring of organs at risk should also be incorporated, and the clinical target volume (CTV) and planning target volume (PTV) should also be drawn.

## 5. Conclusion

In this study, we successfully proposed and verified a robust automated GTV segmentation method for HNC based on DCNN and dual-center PET-CT images. With multimodality images, both anatomic and metabolic features are extracted automatically and objectively, which contribute to the increased accuracy. The DL algorithm showed good potential in GTV segmentation. All these contributed to the high accuracy and efficiency of our method compared to manual contouring. Our method may be helpful in aiding the clinicians in radiotherapy of HNC; thus, it is of great potential in HNC patient management. Future studies may aim to improve further the segmentation accuracy with more training data and optimized network structure, to draw CTV/PTV, and to verify our method with data from multicenters.

## Figures and Tables

**Table 1:** Comparison of the segmentation performance in Experiment 2 between our proposed CNN model and the similar studies.

Studies	Algorithm	Images used	Average DSC	Automatic	Patient number	Center number	Journal
Yang et al. [13]	MRFs	PET, CT, and MRI	0.740	Fully automatic	22	1	Medical Physics
Stefano et al. [14]	AK-RW	PET	0.848	Semiautomatic	18	1	Medical & Biological Engineering & Computing
Berthon et al. [15]	Decision tree	PET	0.770	Fully automatic	20	1	Radiotherapy & Oncology
Song et al. [17]	Graph-based cosegmentation	PET	0.761	Semiautomatic	2	1	IEEE Transactions on Medical Imaging
Zeng et al. [16]	Active surface modeling	PET	0.700	Fully automatic	2	1	Computers in Biology and Medicine
Proposed method	CNN	PET and CT	0.736	Fully automatic	22	2	—

Note: DSC, dice similarity coefficient; MRFs, Markov random fields; AK-RW, adaptive random walker with k-means; CNN, convolutional neural network.

**Table 2:** The segmentation performance of all tumors by using the proposed method in Experiment 2.

Patient number	GTVm (cm^3^)	GTVa (cm^3^)	Sensitivity	Precision	DSC	MSD (mm)
*Database 1*						
1	73.1	61.4	0.619	0.736	0.683	4.8
2	10.4	8.9	0.759	0.886	0.818	2.1
3	3.5	3.7	0.689	0.653	0.670	4.1
4	14.5	16.9	0.832	0.715	0.769	2.6
5	25.9	22.6	0.736	0.844	0.786	2.5
6	27.1	25.7	0.770	0.812	0.791	3.7
7	11.8	11.6	0.713	0.723	0.718	7.0
8	37.5	28.1	0.422	0.562	0.482	10.3
9	65.7	69.5	0.841	0.794	0.817	2.9
10	13.3	12.1	0.534	0.588	0.560	11.5
11	26.7	26.3	0.787	0.799	0.793	1.8
12	17.6	13.5	0.601	0.783	0.680	8.0
13	44.0	44.5	0.873	0.863	0.868	1.2
14	51.8	48.2	0.788	0.847	0.817	2.5
15	73.1	58.2	0.729	0.915	0.812	2.5
16	147.5	147.1	0.782	0.784	0.783	2.5
17	12.1	14.4	0.820	0.692	0.750	2.6
Mean ± SD	—	—	0.723 ± 0.119	0.764 ± 0.100	0.741 ± 0.100	4.2 ± 3.1

*Database 2*						
1	24.5	30.7	0.594	0.475	0.528	13.1
2	80.4	74.0	0.791	0.859	0.824	1.7
3	18.3	20.6	0.918	0.815	0.863	2.9
4	18.2	17.3	0.772	0.815	0.793	6.7
5	69.8	43.0	0.478	0.775	0.591	6.7
Mean ± SD	—	—	0.711 ± 0.174	0.748 ± 0.155	0.720 ± 0.150	6.2 ± 4.5

Note: GTVm, the gross tumor volume of manual delineation; GTVa, the gross tumor volume of automatic segmentation by the proposed method; DSC, dice similarity coefficient; MSD, mean surface distance.

## Data Availability

The authors do not have permission to share data.
